# Design Justice in Action: Co-Developing an HIV and Substance Use Linkage Intervention with Young Adults Involved in the Carceral System

**DOI:** 10.3390/socsci15010055

**Published:** 2026-01-22

**Authors:** Sheridan Sweet, Nicole McCaffery, Jerry Jiang, Robert W. S. Coulter, James E. Egan, Janet Myers, Martha Shumway, Marina Tolou-Shams, Emily F. Dauria

**Affiliations:** 1 Department of Behavioral and Community Health Sciences, School of Public Health, University of Pittsburgh, Pittsburgh, PA 15261, USA; 2 Division of Prevention Science, Department of Medicine, University of California, San Francisco, CA 94158, USA; 3 Department of Psychiatry and Behavioral Sciences, School of Medicine, University of California, San Francisco, CA 94143, USA; 4 Department of Psychiatry, Zuckerberg San Francisco General Hospital and Trauma Center, University of California, San Francisco, CA 94110, USA

**Keywords:** Design Justice, implementation science, community-engaged research, young adults, criminal legal system

## Abstract

To redress systemically biased approaches to health interventions and service design, it is critical that public health researchers employ frameworks that are intentional in their approach to recognizing and working against existing power structures to advance equity in public health. Design Justice represents an approach to design which centers marginalized people and uses collaborative design processes to address community needs and challenges. The purpose of this paper is to describe our process for applying a Design Justice framework to Project XX. Project XX is a study funded by XX designed to develop and test an eHealth-enhanced peer navigation intervention to improve engagement in substance use and HIV-related services for young adults with recent carceral system involvement. We situate the project within the theoretical foundation of Design Justice and community-engaged research, describe its development and implementation, and analyze the application of Design Justice principles from an implementation science perspective by overlaying them with Stanford University’s Center for Dissemination and Implementation’s five key dimensions of dissemination and implementation methods. We highlight successes, challenges, and lessons learned, offering recommendations to guide more equitable and inclusive approaches for future research and practice.

## 1. Introduction

Contact with the carceral system (CS) is closely linked to heightened risk for both HIV acquisition and substance use disorders (Dailey et al. 2020; Maruschak 2022; Widra 2024). Young adults (ages 18–29) are disproportionately represented among individuals who are incarcerated (Carson 2022), and they also shoulder an unequal share of new HIV diagnoses (Dailey et al. 2020). Despite this urgent need, few evidence-based interventions exist to effectively address these intersecting health challenges (Dauria et al. 2022). Young adults (ages 18–29) are disproportionately represented among individuals who are incarcerated in detention settings (Carson 2022), and they also shoulder an unequal share of new HIV diagnoses (Dailey et al. 2020). Despite this urgent need, few evidence-based interventions exist to effectively address these intersecting health challenges (Dauria et al. 2022). To move the field forward, public health researchers must adopt frameworks that not only acknowledge but also actively work to dismantle entrenched power structures that perpetuate inequities. One such framework is Design Justice, which centers the voices of marginalized communities and emphasizes collaborative design processes to create interventions that are both responsive to community needs and sustainable in practice (Costanza-Chock 2020). In this *Commentary*, we apply a Design Justice framework to review the processes used in Project XX, an intervention study designed to address substance use and HIV inequities among young adults (ages 18–29) impacted by the CS. We begin by providing background on health inequities in HIV and substance use among individuals affected by the CS, situating the need for interventions like Project XX. We then outline the theoretical foundations of Design Justice and its connections to community-engaged research. Next, we describe the development and implementation of Project XX, highlighting moments where participatory design practices were effectively integrated as well as areas where challenges in applying participatory approaches persisted. We also briefly discuss the ways in which we analyzed our application of the Design Justice principles from an implementation science perspective in response to these challenges, with the goal of identifying strategies to improve our processes in future work. We conclude our *Commentary* by offering a process for incorporating a Design Justice-guided iterative review of study procedures, reflecting on lessons learned, and suggesting recommendations for future research and practice. The goal of this work is to advance more equitable, inclusive, and responsive approaches to addressing health inequities among multiply marginalized populations, particularly those impacted by the CS.

## 2. Background

### 2.1. HIV, Substance Use, and the Carceral System

The United States (US) has one of the highest incarceration rates in the world (Widra and Herring 2021). Over 5.5 million individuals are under control of the CS, either through confinement in jail or prison, or through community supervision (probation and parole) (Sawyer and Wagner 2023). The CS disproportionately impacts already systemically marginalized groups, particularly those who are Black or Latinx (Nellis 2021), and members of sexual and gender minoritized (SGM) groups (Meyer et al. 2017; Nellis 2021). These disparities in incarceration rates are largely driven by institutional and social biases, contributing to over-policing and the resulting destabilization of these communities (Gaynor 2018; Hinton and Cook 2021).

Individuals who have contact with the CS experience elevated rates of HIV and substance use disorder (SUD) when compared to individuals without CS experience. The prevalence of HIV among incarcerated individuals is 3.7 times higher than in the general US population (Dailey et al. 2020; Maruschak 2022) and the rate of substance use disorder (SUD) is 6.1 times higher (Widra 2024). These disparities stem from structural inequities that increase CS contact among groups already disproportionately affected by HIV and SUD, including people who experience homelessness, engage in sex work, have an SGM identity, or hold a racial or ethnic minoritized identity (Gaynor 2018; Hinton and Cook 2021).

Disparities related to HIV and SUD among individuals with CS contact are further exacerbated by the intersecting impacts of incarceration on other social determinants of health, such as stigma, social support, access to housing, and employment (Cao et al. 2025), impacting healthcare engagement and outcomes across individual, social, and structural levels. This is particularly salient for young adults as they navigate their transition to adulthood and develop skills, attitudes, and behaviors that impact health across their lifespan (Rod et al. 2025).

### 2.2. Young Adults with Carceral System Involvement

Young adults are disproportionately represented in HIV incidence (Dailey et al. 2020), estimated SUD prevalence (SAMHSA 2019, 2022), and incarceration rates (Carson 2022; Howden and Meyer 2011). This heightened vulnerability stems, in part, from developmental and social factors unique to this age group, including ongoing brain maturation into the late twenties, heightened susceptibility to peer influence, identity exploration, and life instability (SAMHSA 2019; Siringil Perker and Chester 2021). These factors illustrate the need for tailored interventions that address the intersecting challenges of young adulthood, CS involvement, HIV, and SU. Yet, few evidence-based interventions currently exist that effectively target these disparities among young adults with carceral system involvement (YA-CSI) (Dauria et al. 2022). Traditional research and program design too often exclude the very populations most affected, leading to solutions that fail to resonate or address structural barriers (George et al. 2015).

### 2.3. Community-Engaged Research: Applications and Challenges

Participatory and co-design approaches offer one pathway for developing interventions and evaluating them for effectiveness in culturally and developmentally relevant ways. These approaches also foster trust in between communities historically marginalized by both health and legal systems and intervention developers (Alang et al. 2021; Attygalle 2017). These approaches share a commitment to engaging participants and system partners in problem definition, solution generation, and refinement, with the goal of improving the relevance, usability, and uptake of interventions with the most promise of solving health problems (Alang et al. 2021; Attygalle 2017). Examples include human-centered design, community-based participatory research, and other co-creation models.

The ways in which researchers use community-engaged methods vary (Masterson et al. 2022), and the degree to which community members are involved throughout the research process remains inconsistent (Green and Johns 2019). For some, community involvement is included considered incidental, an approach which often results in superficial evidence of involvement (Colder Carras et al. 2023; Green and Johns 2019). Even when researchers approach their work with the intention of meaningfully involving communities throughout the research process, they often encounter challenges related to power dynamics and structural barriers, such as institutional policies and administrative timelines that hamper intentional and meaningful collaboration (Adler et al. 2022; Arnold et al. 2022; Okoroji et al. 2023). Researchers may also unintentionally reproduce epistemic hierarchies that privilege academic, biomedical, or institutionally sanctioned forms of knowledge while marginalizing community-based, experiential, and contextual ways of knowing. Consequently, these practices can limit whose knowledge is legitimized, whose interpretations shape research decisions, and whose priorities are reflected in study outcomes, even within ostensibly community-engaged projects (Arnold et al. 2022; Steketee et al. 2020). Addressing these issues requires researchers to thoughtfully interrogate their own assumptions, decision-making processes, and definitions of rigor and evidence, and to examine how their methodological and implementation choices may perpetuate inequities in knowledge production. Researchers have begun to identify and address these issues (Daya et al. 2020; Jones et al. 2023), but more work is needed to identify frameworks and develop tools that researchers can use to ensure their work is truly community-engaged throughout all stages of the implementation process.

### 2.4. Design Justice

Emerging from a transdisciplinary and international network of designers, practitioners, and scholars, the Design Justice framework is a response to the limitations of community-involved research approachs up to now. Design Justice offers a community-led alternative to dominant co-design methodologies that may replicate existing power imbalances (Design Justice Network 2025). Building on established co-design traditions, Design Justice explicitly foregrounds power, equity, and epistemic justice throughout the design lifecycle (Costanza-Chock 2020). Rather than focusing solely on participation, the framework interrogates whose knowledge is considered legitimate, who holds decision-making authority, and who benefits from design outcomes. In this way, Design Justice complements existing co-design approaches by embedding equity and accountability as core design principles rather than ancillary considerations, strengthening participatory practices that might otherwise reproduce hierarchies of knowledge and power.

Sasha Costanza-Chock, a scholar, activist, and designer, has been instrumental in articulating the Design Justice framework. Their foundational text, Design Justice: Community-Led Practices to Build the Worlds We Need (Costanza-Chock 2020), examines how design practices can either perpetuate or disrupt existing power structures and provides a roadmap for reimagining design in pursuit of justice. The Design Justice Network, a collective of designers, artists, and community organizers, has further advanced this framework by articulating ten guiding principles intended to ensure that design processes are led by and accountable to marginalized communities (Design Justice Network 2025; Table 1). These principles are intentionally dynamic, allowing the framework to adapt across contexts while remaining grounded in its core commitments.

## 3. Applying Design Justice Principles to Developing and Testing an Intervention to Improve HIV and Substance Use Service Linkage for Young Adults Involved in the Carceral System

### 3.1. Project LYNX Description

Project LYNX is an ongoing project funded by the National Institute on Drug Abuse (NIDA; R34DA054853 REDACTED) with the overall goal of improving access to HIV prevention and SUD-related services for YA-CSI. Project LYNX leverages patient navigation and eHealth technologies, both of which offer evidence-based strategies to address multifaceted barriers to HIV prevention and SUD service linkage for YA-CSI. Patient navigation promotes engagement in care through one-on-one support to reduce barriers (Freeman and Rodriguez 2011). Peer-led models, in which navigators share lived experiences or identities with clients, have demonstrated effectiveness in building trust and mitigating stigma, particularly among marginalized populations (Cunningham et al. 2017; Myers et al. 2018). eHealth approaches can enhance navigation for YA-CSI. For example, eHealth-supported peer navigation has been shown to sustain virologic suppression among individuals released from jail because of the improved access to sensitive services with electronic communication (Hightow-Weidman et al. 2015; Sullivan et al. 2017). Despite their promise, however, eHealth-enhanced navigation has not been evaluated for linking YA-CSI in the community to integrated HIV prevention and SU services. To address this gap, Project LYNX was undertaken and aimed to: (1) adapt an existing evidence-based navigator model and incorporate codeveloped eHealth technology to refer and link YA-CSI to HIV-prevention (PrEP) and SU services; (2) refine and test the adapted eHealth enhanced, navigator-led SU and HIV prevention (PrEP) service intervention for YA-CSI, for appropriateness and satisfaction and; (3) assess the feasibility, acceptability, and impact of the adapted, eHealth enhanced, navigator program to refer and link YA-CSI to SU and PrEP services. At the time of manuscript submission, the study team is preparing to pilot test the eHealth-supported navigation services.

### 3.2. Project LYNX Community Advisory Board Processes

Beginning in January 2023, as part of a landscape analysis, the research team held 18 meetings with representatives from 15 local organizations that work with YA-CSI. Through these meetings, individuals were identified and invited to apply to a Community Advisory Board (CAB). Our CAB comprised N = 11 individuals with relevant lived experience in the CS, and professional experience providing HIV, SU, and other services to YA-CSI. The first CAB meeting was held in July of 2023.

The CAB met throughout the study and gave feedback on study activities, including: mapping the cascade of HIV-prevention and SU services for YA-CSI in the study setting, co-developing the eHealth intervention enhancement, reviewing recruitment materials and processes and data collection tools, and providing feedback on dissemination strategies. The CAB co-developed community agreements, and CAB meetings were facilitated by members of the research or design teams. CAB members were compensated with a $20 gift card for each one-hour meeting they attended. The full study protocol has been published, providing additional detail about the study procedures (Creasy et al. 2024).

### 3.3. Procedures

During study implementation, we encountered challenges in meaningfully identifying and engaging the communities we sought to partner with, thus impacting both the pace and quality of engagement throughout the study. Although the intervention was initially designed collaboratively with YA-CSI and relevant system partners, the project was disrupted when the Principal Investigator transitioned to a new institution, requiring the study to be re-established within a new organizational and community landscape. In addition, the core research team did not possess lived experience related to the study’s focal areas, which created challenges in authentically engaging YA-CSI and contributed to difficulties in recruiting participants and sustaining CAB member involvement. Maintaining consistent engagement was further complicated by misalignment between the goals and operational priorities of the CS and those of the study team, as well as by turnover among key partners, including community-based organization representatives and individuals with lived experience of incarceration. Finally, coordinating CAB participation proved challenging due to the differing schedules and constraints of frontline healthcare workers, program administrators, community members, and academic team members, further limiting sustained and equitable participation.

To address these issues, we conducted a structured review of our study implementation processes and procedures, grounded in Design Justice principles and guided by a dissemination and implementation (D&I) framework: Stanford University’s Center for Dissemination and Implementation’s five key dimensions of D&I methods (McGovern et al. 2024). We selected this framework because it provides a comprehensive, systems-oriented structure for examining how interventions are designed, implemented, adapted, and positioned for sustainability across complex real-world settings. The Design Justice framework offered a complementary, values-driven lens for critically examining how design decisions shape access, participation, and outcomes, particularly for populations disproportionately affected by health inequities. Its explicit attention to power, knowledge production, and accountability made it well-suited for interrogating and refining our research practices to ensure that the intervention was acceptable and responsive to community priorities.

**Figure 1.**
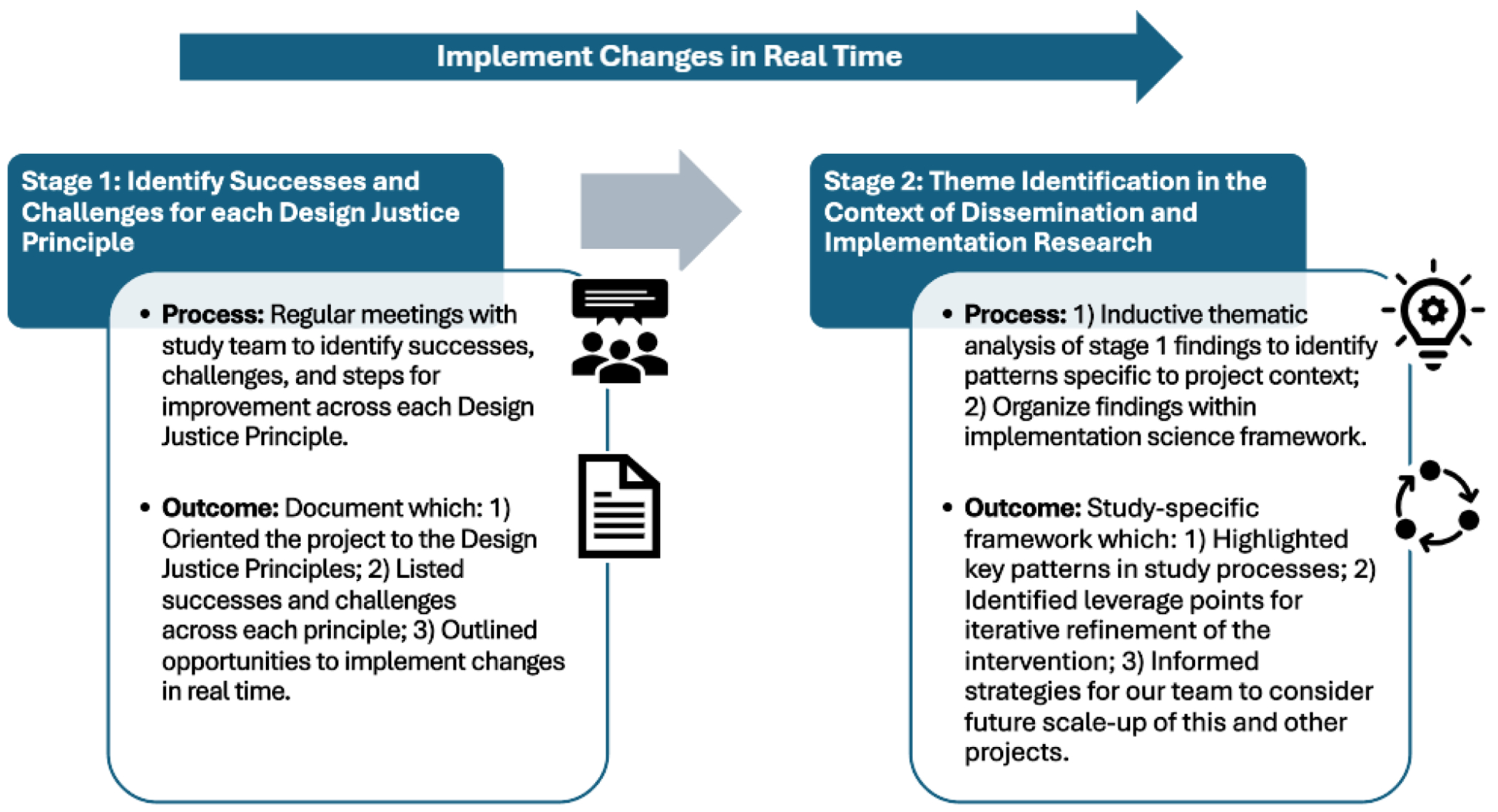
Process for Integrating a Design Justice Framework to Drive Intervention Development, Implementation, and Refinement for Project LYNX.

Our process occurred between January 2023 and June 2025 and included two stages in which our team: (1) identified successes and challenges across each of the ten Design Justice principles, and (2) synthesized cross-cutting themes and organized them to align with the five key dimensions of D&I methods (Figure 1). These stages and brief descriptions of their application and outcomes are outlined in further detail below. Information generated through this analysis was used to identify targeted, actionable steps to adapt study processes and procedures to more effectively center YA-CSI.

Stage 1: Identify Successes and Challenges for each Design Justice Principle. An Excel spreadsheet was used as a structured tool to document and track key observations that our team experienced throughout the intervention development process. For example, the Excel file was used to document the study team’s experiences meeting with study collaborators, engaging with the CAB, recruiting, and collecting pilot data. Separate columns were created to record successes, challenges, and recommendations, organized by Design Justice principle. This format allowed for real-time documentation by project staff during their work (e.g., resulting from team meetings, field work, data collection). Table 2 provides an illustrative example from this spreadsheet. Three people from the study team, two graduate students and the study’s Principal Investigator, held monthly 1 h meetings to review the entries and update them to ensure accuracy and comprehensiveness. The spreadsheet facilitated pattern recognition and informed iterative adaptations to the study implementation by providing a centralized, accessible record of evolving insights. These meetings also provided a platform through which to discuss how these reflections could inform intervention processes in real-time and to develop a plan for implementing these revisions. Following the description of stage 2, we present a case example from Project LYNX, illustrating how we reflected on and refined our co-design practices with our technology partner, through the lens of the Design Justice framework.

Stage 2: Theme Identification in the Context of Dissemination and Implementation Research. In addition to using the study team’s reflections to refine study procedures, we also sought to identify broader patterns in the barriers and facilitators our team encountered to inform our future work. Guided by established thematic analysis principles, we conducted an inductive thematic analysis of the successes and challenges documented in Stage 1 (Braun and Clarke 2006). One study team member independently reviewed the compiled data and generated an initial set of preliminary themes. These themes were then iteratively reviewed, discussed, and refined during weekly meetings with a two-person analytic team until consensus was achieved. To further structure and contextualize these themes, members of the study team organized findings using Stanford University’s Center for Dissemination and Implementation’s five key dimensions of D&I methods (McGovern et al. 2024). These five dimensions are: (1) evidentiary support for the intervention, program, or service; (2) partner engagement; (3) contextual determinants; (4) implementation and sustainment strategies; and (5) implementation and sustainment outcomes. Table 3 outlines a detailed description of each of these dimensions. Beginning in November 2024, our team applied the first four dimensions to develop a deductive coding schema to classify successes and challenges (Stage 1). We omitted the fifth dimension, “implementation and sustainment outcomes”, as this did not align with where we were in the study implementation. Coding discrepancies were resolved through team discussion to reach consensus.

Using an integrated inductive–deductive approach, we developed a study-specific framework that revealed key patterns in study processes, identified leverage points for iterative intervention refinement, and helped outline a strategic plan for scaling this and future projects. Applied in tandem with the Design Justice framework, this approach ensured that our processes remained community-centered and equitable while adhering to best practices in D&I science. It generated actionable insights that guided decision-making across the study lifecycle, supporting both the refinement of the intervention and its broader implementation potential.

### 3.4. Study Team Reflections from Implementing Stages 1 and 2

Stage 1 Case Example: Collaborative Design and Reflexive Engagement in a Community-Engaged Research–Technology Partnership.

Partnership Development and Design Justice Orientation. During the planning phase of Project LYNX, our research team engaged local partners to shape the project’s direction, priorities, and infrastructure. Feedback from these partners informed the selection of a technology collaborator who had experience in participatory technology development and a stated commitment to iterative and collaborative design processes. The resulting grant proposal for Project LYNX, rooted in equity and co-creation in technology intervention development, was funded with this partnership in place.

At the project’s onset, we met with the technology partner to clarify their involvement in the design of the eHealth application and communicated our intent to approach design activities through a Design Justice framework. We explicitly stated our commitment to centering community voices and ensuring that design processes reflected the stated needs and desires of the study population. The technology partner acknowledged and emphasized their interest in supporting this approach.

Reflect-and-Refine Process: Examples from Design Sessions. The CAB participated in multiple design sessions co-led by members from the research and technology teams. During an early session, the research team observed misalignments between the technology partner’s approach and our intended goals. The examples and language used were often inaccessible to CAB members and, at times, potentially harmful. For instance, the technology partner introduced a journey mapping exercise by referencing experiences such as selecting a home mortgage—an example disconnected from the realities of many individuals recently released from incarceration. In addition, the use of stigmatizing, identity-first language (e.g., “inmate”) rather than person-first language (e.g., “individual who has been incarcerated”) risked alienating participants. Although the research team corrected this language in real time, these examples underscored the importance of ensuring inclusive and contextually relevant facilitation to support meaningful CAB engagement.

Immediately following the CAB meeting, the research team convened internally to reflect on the meeting proceedings and to identify potential opportunities to improve processes moving forward. To facilitate the research team’s ability to identify potential improvements, the team reviewed the Design Justice principles, and identified three of particular import: center the voices of those who are directly impacted by the outcomes of the design process (Design Justice Principle #2); see the role of the designer as a facilitator rather than an expert (Design Justice Principle #5); and view change as emergent from an accountable, accessible, and collaborative process rather than a point at the end of the process (Design Justice Principle #4).

Following the internal research team meeting, we engaged the technology partner in a feedback session where we shared detailed observations, highlighted specific moments where harm could have occurred, and offered suggestions for making future sessions more inclusive, transparent, and aligned with Design Justice principles. We also acknowledged that the research team should have provided background information and resources from the outset to better support the technology partner in facilitating with sensitivity to community context. To address this gap, we created a reference document outlining preferred terms and those to avoid and shared additional informational resources. The technology partner was receptive, adjusting their facilitation practices to better reflect community values. Subsequent design sessions were markedly improved. CAB members expressed greater comfort and clarity, and the tone of engagement shifted to one that more authentically reflected shared power and respect for lived experience. The success of this exchange may be attributed, in part, to our use of Design Justice Principle #4, which emphasizes change as an accessible and collaborative process rather than a discrete endpoint. Our team continues to focus on improving collaborative design by thinking critically about design processes and intentionally expanding them. This includes maintaining an iterative feedback loop with our technology partner, where we collaboratively examine how design decisions are presented, who facilitates them, and how well they resonate with and reflect the community’s voice.

Challenges and Lessons Learned. This experience reminded us that trust in collaborators must be balanced with accountability. Despite shared intentions and initial verbal agreements, our team encountered challenges in upholding these principles in practice. Early in the collaboration, we deferred too heavily to the technology team’s design expertise, trusting that their standard facilitation practices would align with our project values. In doing so, we inadvertently offloaded responsibility for shaping the tone and structure of design sessions, missing early opportunities to ensure that community-centered values were upheld.

Stage 2: Sample Findings and Reflections.

We identified 28 successes and 27 challenges in implementing Project LYNX across all the Design Justice principles. After organizing our findings according to Stanford University’s Center for Dissemination and Implementation’s five key dimensions of D&I methods (McGovern et al. 2024), we identified distinct patterns across each domain.

The highest number of challenges was observed in the *Contextual Determinant* dimension. Administrative and institutional factors presented the most significant barriers to aligning our work with Design Justice principles. For example, grant structures and deadlines pushed the project forward, even when the community had not yet been fully and meaningfully integrated into intervention design processes. We identified our teams’ biggest successes in applying the Design Justice principles in the *Planning for Partner Engagement* dimension: eight successes and one challenge were identified across six Design Justice principles. For example, to center people with lived and living experience in SU, HIV, and the CS, we operated with transparency regarding our data use and sharing, giving folks the opportunity to choose if they want to participate based on this, while also offering a safer way for them to share their experiences without fear of legal or social repercussion.

We identified 16 successes and 12 challenges across all 10 Design Justice principles in the *Implementation and Sustainment Strategies* dimension. Many of our challenges in this dimension were related to our CAB processes, including meeting structure and facilitation, which we allowed to be determined largely by our tech partner. This was further exacerbated by pressure to meet grant requirements and deadlines, which made reflecting on processes and reiterating upon them in real time challenging. Many of our successes in the adaptation phase of this dimension were processes we implemented in response to the challenges that emerged. Notably, we altered the structure of our CAB meetings early on as engagement dwindled to allow for multiple points of flexible participation through large group meetings, scheduled one-on-one meetings with a team member, and CAB “drop-in” hours.

Across the development and implementation of Project LYNX, the team identified nearly equal numbers of successes (28) and challenges (27) in applying Design Justice principles, offering critical insights to guide future practice. While administrative and institutional constraints—such as grant timelines—posed barriers to fully centering community input, the process also revealed clear strategies for improvement. Our strongest alignment with Design Justice principles occurred in the Planning for Partner Engagement dimension, where transparency, choice, and safer participation opportunities strengthened collaboration with people with lived and living experience. Challenges in Implementation and Sustainment, particularly around CAB processes, prompted adaptive changes such as restructuring meetings to increase flexibility and engagement. The processes outlined in this stage allowed us to identify specific areas of strength and improvement along with the implementation of this research study to identify the timing and source of challenges and facilitators, and potential levers to implement changes to address the barriers that we experienced. This influenced our processes by allowing us to focus on places where we were able to make changes and better communicate with partners about things that were outside of our control.

Challenges and Lessons Learned. Given the barriers we experienced in engaging with our CAB, CAB members were not integrated into the reflection activities, which were conducted retrospectively. As a result, we cannot be certain that the successes and challenges identified, or their alignment within the D&I framework, fully reflect the CAB’s perspectives and lived experiences. Additionally, this effort was not intended as a comprehensive scientific analysis, but rather as a process-focused reflective activity to examine implementation experiences. Embedding these reflection and analytic processes prospectively, with intentional CAB involvement, may enhance rigor and relevance in future work.

## 4. Discussion

Our experience demonstrates that the Design Justice model offers researchers an actionable and concrete strategy for ensuring that a community’s priorities remain centered in the development of interventions, and programs, with the aim of creating solutions that are both contextually appropriate and effective. Integrating an implementation science lens strengthens this application by examining how interventions are delivered, adapted, and sustained. This combined perspective underscores the importance of embedding reflexivity, shared power, and community accountability throughout the full lifecycle of research and practice partnerships, while also attending to implementation determinants, processes, and outcomes. Even in collaborations that begin with strong alignment, tensions and challenges can arise (Arnold et al. 2022; Egid et al. 2021). Approaching these moments through a Design Justice lens with attention to the implementation stage not only helps address immediate concerns but also enhances rigor, transparency, and generalizability by linking equity-centered practices to established implementation frameworks and analytic strategies.

### Recommended Process: Design Justice-Guided Iterative Review of Study Processes

Drawing from the experiences on Project LYNX, we encourage research teams to adopt a Design Justice–guided, collaborative process to support ongoing reflection, review, and refinement of study procedures throughout the implementation period. The recommended process operationalizes Design Justice principles by embedding structured, recurring opportunities for research team members and CAB members to jointly assess study processes, identify barriers and facilitators, and co-develop responsive implementation adaptations.

#### Participants and Roles.

This process should engage two groups: (1) the core research team, including investigators and project staff, and (2) the CAB, comprising individuals representing communities most impacted by the study focus and implementation partners. CAB members should be compensated for their time and positioned as equal partners in decision-making related to study procedures. The research team should be responsible for facilitating review activities, maintaining documentation, and ensuring that collaboratively identified refinements are translated into actionable protocol updates that are accessible to all participating groups.

#### Potential Data Sources for Iterative Review.

To inform iterative reflection and refinement, teams could draw on multiple data sources, including: (1) implementation reflections documented by research staff following key study activities (e.g., recruitment, data collection, intervention delivery); (2) CAB feedback captured during structured meetings and ongoing communication; (3) process tracking tools, such as implementation logs or issue trackers maintained across the project period. These materials should be compiled in a centralized tracking system to support transparency and longitudinal review.

#### Iterative Review Cycle.

Teams are encouraged to conduct Design Justice–guided review cycles at regular intervals and/or in alignment with key implementation milestones (e.g., drawing from, for example, Stanford University’s Center for Dissemination and Implementation’s five key dimensions of D&I methods). An example of a cycle using this framework could include five core components:

1. Preparation and Synthesis. Prior to each review session, the research team should synthesize recent implementation reflections and process notes, highlighting salient successes, challenges, and decision points. These materials should be prepared in ways that support continuity and onboarding (e.g., in the event of CAB turnover), including clear documentation of prior decisions and rationales. Key items should be explicitly mapped to relevant Design Justice principles (e.g., centering community leadership, minimizing harm, equitable distribution of benefits) and implementation science frameworks using accessible language and formats to ensure meaningful engagement by CAB members with varied lived experiences, roles, and levels of familiarity with research and implementation science concepts.
2. Collaborative Review Sessions. Research team members and CAB members should convene in structured review sessions facilitated using guided prompts or activities. These prompts should encourage participants to assess the extent to which study processes align with Design Justice principles and to identify areas requiring adjustment, and note where in implementation processes these could be addressed. CAB members should be supported to validate or contradict interpretations, raise concerns, and propose community-informed alternatives.
3. Co-Development of Adaptations. Proposed adaptations to study procedures should be collaboratively generated during review sessions, with explicit attention to feasibility, ethical implications, and potential unintended consequences. Decision-making should prioritize CAB perspectives, particularly for changes that affect participant burden, accessibility, trust, or community benefit.
4. Documentation and Accountability. All agreed-upon adaptations should be documented in a shared action log that specifies the rationale for changes, responsible parties, and anticipated timelines. Teams should communicate back to CAB members about how recommendations were implemented, modified, or deferred to reinforce transparency and accountability.
5. Sustainability and Continuous Improvement. Teams should periodically reflect on the effectiveness of this iterative review process itself. This reflection should include structured opportunities for CAB members to provide feedback on the extent to which power sharing, transparency, and collaborative decision-making were meaningfully enacted. Teams are encouraged to assess whether the review cadence, facilitation approaches, and documentation practices adequately support equitable participation and timely responsiveness to community-identified concerns. Based on this feedback, teams should refine review structures as needed and document recommendations for sustaining Design Justice–aligned practices beyond the current study, including their integration into future projects, institutional workflows, or partnership agreements.

#### Cross-Cycle Learning and Synthesis.

Across iterative review cycles, teams are encouraged to conduct an inductive synthesis of documented barriers, facilitators, and adaptations to identify cross-cutting patterns in implementation experiences. These analyses can inform both real-time decision-making and longer-term learning about how Design Justice principles shape study processes over time. CAB members should be engaged in reviewing and refining emergent themes to ensure interpretations reflect community perspectives.

#### Ethical Considerations and Power Sharing.

This recommended process is intended to move beyond consultative engagement by embedding CAB members in ongoing implementation decision-making. Power sharing should be reinforced through transparent documentation, shared agenda setting, equitable compensation, and explicit recognition of CAB expertise. The iterative nature of the process allows teams to respond to emerg ing concerns in ways that reduce harm, increase trust, and strengthen alignment with community priorities.

#### Potential Deliverables.

Teams implementing this recommended process may choose to develop additional deliverables to support transparency, dissemination, and sustainability. These may include a Design Justice mapping table that links specific implementation issues to relevant Design Justice principles and corresponding actions, which can serve as both an analytic and accountability tool. Teams may also create a visual diagram of the iterative review cycle to facilitate shared understanding among research staff, CAB members, and external stakeholders. Summary memos or infographics synthesizing key refinements and lessons learned can be prepared for funders, institutional leadership, or community partners to document the value of the process and inform decision-making. Finally, teams may include a description of the iterative review process and associated tools as a supplementary appendix in manuscripts or reports to support replication by other research teams.

Unlike more traditional approaches that engage CAB for discrete or episodic tasks (e.g., troubleshooting recruitment challenges, reviewing specific study materials), this recommended process positions CAB members as ongoing partners in the continuous examination and refinement of study implementation. By embedding CAB engagement within recurring, structured review cycles, the process shifts CAB involvement from reactive consultation to proactive, longitudinal collaboration. This approach allows community perspectives to shape not only isolated decisions but also the evolving norms, priorities, and procedures of the project across its full implementation lifecycle. In doing so, it creates sustained opportunities for shared learning, reinforces accountability to Design Justice principles, and supports deeper, more durable CAB engagement by recognizing community expertise as essential to adaptive implementation rather than as an ancillary input at select moments.

## 5. Conclusions

Our experience highlights the need to further examine how frameworks such as Design Justice can be operationalized in dissemination and implementation science and community-engaged research. Future scholarship, building off of the emergent literature (e.g., (McGladrey et al. 2025)), can deepen understanding of the mechanisms through which reflexive, equity-centered practices influence partnership dynamics, decision-making, and implementation outcomes, while also identifying pragmatic strategies to evaluate and measure their impact using established D&I constructs.

Beyond research, the Design Justice framework also has important implications for public health practice. When paired with the D&I framework applied in this study, it supports practitioners in moving beyond community consultation toward authentic co-creation of programs, systems, and policies, while also attending to feasibility, scalability, and sustainability in real-world settings. Importantly, as our work demonstrates, Design Justice can be integrated at any phase of a project, and a D&I lens helps structure these moments of reflection, realignment, and reprioritization to ensure that evolving goals and practices remain grounded in community needs, equity principles, and implementation realities. Ultimately, integrating Design Justice into public health practice shifts the focus from delivering services to transforming systems; thus, creating more equitable, sustainable, and community-driven pathways to health and wellbeing.

## Figures and Tables

**Table 1.** Design Justice Principles and Description.

Principle	Description
**Principle 1**	We use design to **sustain, heal, and empower** our communities, as well as to seek liberation from exploitative and oppressive systems.
**Principle 2**	We **center the voices of those who are directly impacted** by the outcomes of the design process.
**Principle 3**	We **prioritize design’s impact on the community** over the intentions of the designer.
**Principle 4**	We view **change as emergent from an accountable, accessible, and collaborative process**, rather than as a point at the end of a process.
**Principle 5**	We see the role of the **designer as a facilitator rather than an expert**.
**Principle 6**	We believe that **everyone is an expert based on their own lived experience**, and that we all have unique and brilliant contributions to bring to a design process.
**Principle 7**	We **share design knowledge and tools** with our communities.
**Principle 8**	We work towards **sustainable, community-led and -controlled** outcomes.
**Principle 9**	We work towards **non-exploitative solutions** that reconnect us to the earth and to each other.
**Principle 10**	Before seeking new design solutions, **we look for what is already working** at the community level. We honor and uplift traditional, indigenous, and local knowledge and practices.

**Table 2.** Illustrative Example of Identified Successes and Challenges from Project LYNX Organized by Design Justice Principle.

Design Justice Principle	Where Have We Been Challenged with This Principle?	Where Have We Had Success Applying This Principle?	How Could We Improve Our Process?
Design Justice Principle Number 1: We use design to sustain, heal, and empower our communities, as well as to seek liberation from exploitative and oppressive systems.	Because we are working to improve conditions within an oppressive criminal legal system, part of the challenge is to do so without feeding resources into harmful system or otherwise legitimizing the existing system.	We focus on empowerment by conceptualizing the goal of substance use treatment linkage within the context of harm reduction.	Hiring practices & improving representation on study team
	We are reckoning with structural inequities while also trying to produce a product/provide an intervention—the timeline required to address the former is much longer than the time alloted to do the latter.	We engage in self-reflexivity (being transparent about our identities during project outreach and partnership development)	Continued community engagement
	The research/design team does not have the direct lived experience of criminal legal involvement and so the healing and empowerment process is separated from those most directly affected.	We explicitly name systemic harms as part of work in this space, describing where & how they shape disparities in all spaces in which we operate, to maintain the tension of improving conditions without feeding the oppressive system.	
		In line with empowerment of communities, the project involves staffing & budget for jobs uniquely developed for peers which will be implemented with Aim 2	
		We engage and collaborate with organizations & people (systems partners) with similar goals/values	

**Table 3.** Descriptions of Stanford University’s Center for Dissemination and Implementation’s Five Key Dimensions of Dissemination and Implementation Methods.

Dimension	Description
Evidentiary Support	The data that are used to support the development and implementation of the intervention/program/service.
Partner Engagement	Planning for the degree to which key and representative partners from the project site(s) and the community in which the project takes place had input into defining the population, clinical problem, or intervention/program/service being delivered to help ensure that interventions will be effective across diverse groups/contexts and used and sustained in practice over time.
Contextual Determinants	Consideration of systems, organizational, provider, and patient/consumer-level factors that affect implementation of the intervention (i.e., barriers and facilitators) that may impact the reach and adoption of the intervention/program/service.
Implementation Strategies	Selection, adaptation, and description of, methods, activities, and resources that support implementation and sustainment of the intervention/program/service (e.g., training, facilitation/coaching, incentives, performance data, audit and feedback), operationalized as the steps and methods taken to support users (within the project or in the real world) with the installation or sustainment of the intervention/program/service.
Implementation Outcomes	Evaluation of effects of actions to implement or sustain the intervention, program, or service (how much and how well an intervention was implemented/sustained).

## Data Availability

No new data were created or analyzed in this study. Data sharing is not applicable to this article.
